# Core–Shell Interface Engineering Strategies for Modulating Energy Transfer in Rare Earth-Doped Nanoparticles

**DOI:** 10.3390/nano14161326

**Published:** 2024-08-07

**Authors:** Zhaoxi Zhou, Yuan Liu, Lichao Guo, Tian Wang, Xinrong Yan, Shijiong Wei, Dehui Qiu, Desheng Chen, Xiaobo Zhang, Huangxian Ju

**Affiliations:** 1 State Key Laboratory of Analytical Chemistry for Life Science, School of Chemistry and Chemical Engineering, Nanjing University, Nanjing 210023, China; 522022240053@smail.nju.edu.cn (Z.Z.); liuzhuolan@stu.xaau.edu.cn (Y.L.); 522022240036@smail.nju.edu.cn (T.W.); xinrongyan@smail.nju.edu.cn (X.Y.); weishijiong@smail.nju.edu.cn (S.W.); xhgdh@smail.nju.edu.cn (D.Q.); deisenchen@nju.edu.cn (D.C.); 2 School of Pharmacy, Nanjing University of Chinese Medicine, Nanjing 210023, China; 20220849@njucm.edu.cn

**Keywords:** RENPs, synthesis strategy, interface clarity, energy transfer, orthogonal biomedical probes

## Abstract

Rare earth-doped nanoparticles (RENPs) are promising biomaterials with substantial potential in biomedical applications. Their multilayered core–shell structure design allows for more diverse uses, such as orthogonal excitation. However, the typical synthesis strategies—one-pot successive layer-by-layer (LBL) method and seed-assisted (SA) method—for creating multilayered RENPs show notable differences in spectral performance. To clarify this issue, a thorough comparative analysis of the elemental distribution and spectral characteristics of RENPs synthesized by these two strategies was conducted. The SA strategy, which avoids the partial mixing stage of shell and core precursors inherent in the LBL strategy, produces RENPs with a distinct interface in elemental distribution. This unique elemental distribution reduces unnecessary energy loss via energy transfer between heterogeneous elements in different shell layers. Consequently, the synthesis method choice can effectively modulate the spectral properties of RENPs. This discovery has been applied to the design of orthogonal RENP biomedical probes with appropriate dimensions, where the SA strategy introduces a refined inert interface to prevent unnecessary energy loss. Notably, this strategy has exhibited a 4.3-fold enhancement in NIR-II in vivo imaging and a 2.1-fold increase in reactive oxygen species (ROS)-related photodynamic therapy (PDT) orthogonal applications.

## 1. Introduction

Rare earth nanoparticles (RENPs), which include both upconversion and down-shifting variants, have attracted considerable interest for their use in a wide range of advanced applications. In the realm of biomedical imaging, nanoparticles such as quantum dots, silicon nanoparticles, and RENPs can be easily tailored to target various biomarkers in vivo [1]. This versatility makes them extensively applicable in bioimaging. RENPs, in particular, are valued for their capability to deliver high-resolution, non-invasive imaging with minimal background interference, which is crucial for visualizing cellular and molecular processes in vivo [2,3,4,5]. Their unique luminescent properties make them particularly suitable for deep-tissue imaging, thereby enhancing the detection and monitoring of disease conditions [3,4]. In drug delivery, RENPs function as efficient carriers for therapeutic agents, offering targeted release and reducing systemic toxicity [6]. Their adjustable optical properties enable precise control over drug release in response to external stimuli like light, temperature, or pH, thereby boosting treatment efficacy and safety [7,8]. In biosensing, RENPs offer sensitive and selective detection capabilities [9,10,11]. Their large Stokes shift and ability to generate a strong signal in the NIR-II window minimize background autofluorescence and maximize detection sensitivity, making RENPs ideal for developing highly sensitive and specific biosensors for various biological analytes [12,13]. Optical multiplexing utilizes the distinct emission profiles of RENPs to enable simultaneous detection of multiple analytes or imaging modalities [14]. This is particularly useful in complex biological systems, where distinguishing between multiple signals is essential for accurate analysis [15]. In anti-counterfeit technologies [16], RENPs are employed to create complex and secure optical signatures that are difficult to replicate, providing a reliable method for verifying the authenticity of products and documents. Photodynamic therapy (PDT) harnesses the phototoxic properties of RENPs, along with their ability to generate reactive oxygen species upon light activation, to selectively target and destroy cancer cells while preserving surrounding healthy tissue [17,18]. The tunable optical properties of RENPs allow for precise control over the treatment area, optimizing therapeutic outcomes and minimizing side effects.

The luminescence properties of RENPs are greatly affected by the type, concentration, and spatial distribution of dopants within the host lattice. Engineering core–shell structures facilitate the synthesis of luminescent nanoparticles, allowing for precise control over the crystal structure, diameter, morphology, surface characteristics, and optical properties [19]. It is crucial to precisely control the growth of the shell to ensure the desired structure and prevent unwanted crosstalk between different emitters in multifunctional core–shell nanocrystals [20,21]. The orthogonal luminescent applications of multifunctional core–shell RENPs show great promise, with efficient energy transfer control being crucial for their advancement. Encouragingly, advanced synthesis techniques for core–multi-shell structures offer the necessary technical basis for optimizing these applications [22]. A variety of synthetic methodologies can be employed to incorporate distinct lanthanide dopants into the discrete layers of core–shell nanostructures [23], and cation intermixing has been observed at the core–shell interface [24,25]. Increasing attention has been directed towards designing RENPs with multi-shells, either inert or active, to achieve higher luminescence intensity or multiple emissions. 

**Figure 1:**
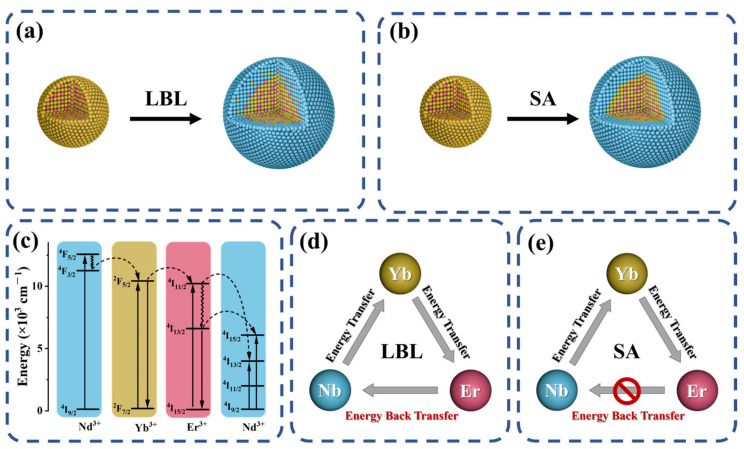
(**a**,**b**) depict the synthesis of RENPs using the LBL strategy, while (**c**) illustrates simplified energy-level diagrams showing the energy transfer between Nd^3+^, Yb^3+^, and Er^3+^ ions upon 808 nm excitation. (**d**,**e**) illustrate the energy transfer between rare earth ions in RENPs-LBL and RENPs-SA, respectively.

High-quality RENPs can be produced using thermal decomposition, hydrothermal/solvothermal processes [26], and microfluidic devices [27]. Thermal decomposition has become a standard method for producing nanoparticles with remarkable uniformity and colloidal stability, which are prerequisites for comprehensive photophysical investigations and applications in sensing and therapy. There are two prevalent synthesis strategies. One is the one-pot successive layer-by-layer (LBL) method proposed by Li et al. [28] (Figure 1a), where a shell’s growth is achieved by alternately injecting shell precursor solutions into the reaction mixture at high temperatures. This successive LBL approach offers advantages over other methods, such as the flexibility to coat multiple shells and the ability to control a shell’s thickness accurately [29]. Another common technique is the seed-assisted (SA) growth method for RENP synthesis (Figure 1b), which typically involves the incorporation of pre-synthesized RENP cores into the reaction medium as seed nuclei prior to the nucleation or incubation of shell precursors [30,31].

However, the slow progress in synthesis technology has led to several unresolved issues, such as understanding the synthesis strategy’s effect on cation mixing. Cation intermixing can occur due to solid-state diffusion, partial dissolution of the core followed by recrystallization in the presence of shell precursors, or a combination of these processes [32]. This interfacial cation mixing is vital as it can modulate energy transfer mechanisms, thereby influencing the emission intensity of the RENPs [33,34]. As illustrated in Figure 1a,b, a distinct interface effectively prevents energy back-transfer between different shell layers containing non-identical rare earth elements (e.g., Er to Nd, as shown in Figure 1c) [32,35,36]. In contrast, the LBL synthesis method, due to element mixing, enhances this energy exchange (though in practical applications, this exchange often results in non-radiative energy relaxation, causing energy loss), as depicted in Figure 1d,e.

To further corroborate and elucidate the aforementioned phenomena, we utilized two refined synthesis techniques—the one-pot successive LBL and the SA strategies—to precisely control the interfacial clarity between the core and shell for studying energy transfer. By consistently maintaining the doping levels of lanthanide elements, solvent ratios, and reaction temperatures across all layers, we isolated the effects of interfacial fusion from the other variables. This standardized approach allowed us to directly link any observed changes in the RENPs’ optical properties to the varying degrees of interfacial integration. The optical characteristics of these RENPs were comprehensively evaluated to determine the impact of interfacial fusion on their energy transfer. Building on these findings, we incorporated the synthesis strategy to propose a rational design for orthogonal RENP probes. Specifically, the SA strategy was utilized to introduce a refined inert interface to mitigate unnecessary energy loss. Notably, this strategy produced significant effects in NIR-II in vivo imaging and reactive oxygen species (ROS)-related PDT orthogonal applications. The results demonstrate that Ce-RENPs-SA nanoprobes exhibit a 4.3-fold enhancement in NIR-II imaging and a 2.1-fold increase in ROS generation. We hope that this research will advance the development of RENPs for high-performance applications in photonics and biomedicine.

## 2. Materials and Methods

### 2.1. Materials

Oleic acid (OA, 90%), 1-octadecene (ODE, 90%), and rose bengal (RB) were purchased from Sigma-Aldrich (Waltham, MA, USA). Anhydrous rare-earth chloride YCl_3_ (99.9%), YbCl_3_ (99.9%), ErCl_3_ (99.9%), GdCl_3_ (99.9%), CeCl_3_ (99.9%), NdCl_3_ (99.9%), TmCl_3_ (99.9%), sodium hydroxide (NaOH), ammonium fluoride (NH_4_F), methanol (MeOH), cyclohexene, N,N-dimethylformamide (DMF), 2-methoxyethanol, 1-ethyl-(3-dimethyllaminopropyl) carbodiimide hydrochloride (EDC), 2-[4-(2-hydroxyethyl)-1-piperazinyl] ethanesulfonic acid (HEPES), N-hydroxy succinimide (NHS), Igepal CO-520, tetraethylorthosilicate (TEOS), and 3-aminopropyl triethoxysilane (APTES) were purchased from Aladin Ltd. (Shanghai, China). NHS-PEG-folate (MW 2000) was purchased from Toyongbio (Shanghai, China). Singlet oxygen sensor green reagent (SOSG) was purchased from Meilun Biotechnology Co., Ltd. (Dalian, China). Human cervix carcinoma (HeLa) cell lines were purchased from Keygen Biotech (Nanjing, China). All other chemical reagents with analytical grade were used directly without further purification.

### 2.2. Composition of RENPs

Tm-RENPs: NaYF_4_: 0.5%Tm, 30%Yb @ NaYF_4_: 10%Yb, 30%Nd

Er-RENPs: NaYF_4_: 2%Er, 20%Yb @ NaYF_4_: 5% Nd

Ce-RENPs: NaYF_4_: 2%Er, 20%Yb, 20%Ce @ NaYF_4_ @ NaYF_4_: 5%Nd

The specific schematic diagram of the structures and the compositions of Tm-RENPs, Er-RENPs, and Ce-RENPs are provided in Figure S1 and Table A1.

### 2.3. Method 1: One-Pot Successive LBL Strategy

This section utilized the core–shell–shell structured Ce-RENPs as an example to describe the one-pot successive LBL synthesis strategy thoroughly. The Tm-RENPs and Er-RENPs were identical to the above nanoparticles, except for the use of different core nanoparticles as seeds and the corresponding lanthanide shell precursors mentioned in Table A1 in Appendix A. 

#### 2.3.1. Syntheses of Ce-RENPs Shell Precursors

YCl_3_ (1 mmol) was mixed with 5.5 mL of OA and 15 mL of 1-octadecene and was heated to 160 °C for 60 min in a vacuum. The reaction solution was then cooled down to 45 °C, dropwise added with a methanol solution of NH_4_F and NaOH (10 mL, molar ratio of 4:2.5), and was continuously stirred for 30 min. The reaction mixture was heated to 110 °C for 15 min to remove the methanol completely and then cooled down to 45 °C to obtain the Ce-RENPs shell 1 precursor. 

The Ce-RENPs shell 2 precursor was prepared according to the same procedure with YCl_3_: NdCl_3_ molar ratio of 95:5. To be specific, YCl_3_ (0.95 mmol) and NdCl_3_ (0.05 mmol) were mixed with 5.5 mL of OA and 15 mL of 1-octadecene and heated to 160 °C for 60 min in a vacuum. The reaction solution was then cooled down to 45 °C. A total of 10 mL of methanol solution of NH_4_F (4 mmol) and NaOH (2.5 mmol) were then added dropwise into the above mixtures and continuously stirred for 30 min. The reaction mixture was heated to 110 °C for 15 min to remove the methanol completely and then cooled down to 45 °C to obtain the Ce-RENPs shell 2 precursor.

#### 2.3.2. Synthesis of Ce-RENPs Core

YCl_3_ (0.58 mmol), ErCl_3_ (0.02 mmol), YbCl_3_ (0.2 mmol), and CeCl_3_ (0.2 mmol) were mixed with 5.5 mL of OA and 15 mL of 1-octadecene and heated to 160 °C for 60 min in a vacuum with subsequent cooling to 45 °C; 10 mL of the methanol solution of NH_4_F (4 mmol) and NaOH (2.5 mmol) were then added dropwise into the above mixtures and continuously stirred for 30 min. The reaction mixture was heated to 110 °C for 15 min and 300 °C for 90 min under a nitrogen atmosphere to obtain the Ce-RENPs core.

#### 2.3.3. Synthesis of Ce-RENPs-LBL Core@Shell 1 with Different Thicknesses of Shell 1

For a layer-by-layer coating process to proceed, the core nanoparticles were treated with different amounts of NaYF_4_ precursor (3 mL added for each layer). The above-prepared isolation shell 1 precursor was injected into the reaction mixture of the core, and 3 mL of the shell 1 precursor was injected at 15 min intervals and kept at 300 °C for 30 min to grow shell 1 on the core (core@shell 1). With the increase in shell 1 injection times, the shell 1 thickness of the Ce-RENPs-LBL core@shell 1 nanoparticles obtained was 1.0 nm, 3.4 nm, 5.3 nm, and 8.1 nm, respectively. The synthesis strategies of Ce-RENPs-LBL with different thicknesses of shell 1 are shown in Figure S2.

#### 2.3.4. Synthesis of Ce-RENPs-LBL Core@Shell 1@Shell 2

Subsequently, the shell 2 precursor was injected into the reaction mixture containing the Ce-RENPs core@shell 1. To be specific, 3 mL of the shell 2 precursor was added at 15 min intervals, maintaining the mixture at 300 °C for 30 min to facilitate the growth of shell 2 on the core@shell 1, resulting in the formation of core@shell 1@shell 2 nanoparticles. The as-prepared Ce-RENPs (core@shell 1@shell 2) were cooled down to room temperature, precipitated with acetone 20 mL acetone, collected by centrifugation at 10,000 rpm for 5 min, subsequently washed twice with a cyclohexane/acetone mixture (*v*/*v* = 1/3), and finally dispersed in 10 mL of cyclohexane for further use.

### 2.4. Method 2: Modified SA Growth Strategy

This section utilized the core–shell–shell structured Ce-RENPs as an example to describe the modified SA strategy thoroughly. Tm-RENPs and Er-RENPs were identical to the above nanoparticles, except for the use of different core nanoparticles as seeds and corresponding lanthanide shell precursors, which were not elaborated upon here.

#### 2.4.1. Synthesis of Ce-RENPs Core

The synthesis of NaYF_4_: Er, Yb, Ce core is consistent with Method 1. To be specific, YCl_3_ (0.58 mmol), ErCl_3_ (0.02 mmol), YbCl_3_ (0.2 mmol), and CeCl_3_ (0.2 mmol) were mixed with 5.5 mL of OA and 15 mL of 1-octadecene and heated to 160 °C for 60 min in a vacuum with subsequent cooling to 45 °C; 10 mL of the methanol solution of NH_4_F (4 mmol) and NaOH (2.5 mmol) were then added dropwise into the above mixtures and continuously stirred for 30 min. The reaction mixture was heated to 110 °C for 15 min and 300 °C for 90 min under a nitrogen atmosphere to obtain the Ce-RENPs core. After naturally cooling down to room temperature, the as-synthesized nanoparticles in the solution were precipitated by adding 20 mL of acetone, collected by centrifugation at 10,000 rpm for 5 min, subsequently washed twice with a cyclohexane/acetone mixture (*v*/*v* = 1/3), and finally dispersed in 10 mL of cyclohexane for further use.

#### 2.4.2. Syntheses of Ce-RENPs Shell 1 Precursor and Shell 2 Precursor

The synthesis of the shell precursors is consistent with Method 1. To be specific, YCl_3_ (1 mmol) was mixed with 5.5 mL of OA and 15 mL of 1-octadecene and heated to 160 °C for 60 min in a vacuum. The reaction solution was then cooled down to 45 °C, dropwise added with a methanol solution of NH_4_F and NaOH (10 mL, molar ratio of 4:2.5), and continuously stirred for 30 min. The reaction mixture was heated to 110 °C for 15 min to remove the methanol completely and then cooled down to 45 °C to obtain the Ce-RENPs shell 1 precursor. 

The Ce-RENPs shell 2 precursor was prepared according to the same procedure with YCl_3_: NdCl_3_, with a molar ratio of 95:5. To be specific, YCl_3_ (0.95 mmol) and NdCl_3_ (0.05 mmol) were mixed with 5.5 mL of OA and 15 mL of 1-octadecene and heated to 160 °C for 60 min in a vacuum. The reaction solution was then cooled down to 45 °C. The reaction solution was then cooled down to 45 °C. A total of 10 mL of the methanol solution of NH_4_F (4 mmol) and NaOH (2.5 mmol) were then added dropwise into the above mixtures and continuously stirred for 30 min. The reaction mixture was heated to 110 °C for 15 min to remove the methanol completely and then cooled down to 45 °C to obtain the Ce-RENPs shell 2 precursor.

#### 2.4.3. Synthesis of Ce-RENPs-SA Core@Shell 1 with Different Thicknesses of Shell 1

The previously prepared Ce-RENPs core cyclohexane solution was added into the Ce-RENPs shell 1 precursor directly and the temperature was raised to 60 °C for 15 min to remove the cyclohexane from the mixture. The reaction solution was then cooled down to 45 °C. The reaction mixture was heated to 110 °C for 15 min and 300 °C for 45 min under a nitrogen atmosphere. The as-prepared Ce-RENPs core@shell 1 was cooled down to room temperature, precipitated with acetone, repeatedly washed with cyclohexane, and redispersed in 10 mL of cyclohexane for further use. Repeating this step, and with the increase in shell1 coating times, the shell 1 thickness of the Ce-RENPs-SA core@shell 1 nanoparticles obtained was 1.2 nm, 3.2 nm, 5.4 nm, and 7.6 nm, respectively. After naturally cooling down to room temperature, the as-synthesized Ce-RENPs-SA core@shell 1 nanoparticles in the solution were precipitated by adding 20 mL of acetone, collected by centrifugation at 10,000 rpm for 5 min, subsequently washed twice with a cyclohexane/acetone mixture (*v*/*v* = 1/3), and finally dispersed in 10 mL of cyclohexane for further use. The synthesis strategies of the Ce-RENPs-SA with different thicknesses of shell 1 are shown in Figure S3.

#### 2.4.4. Synthesis of Ce-RENPs-SA Core@Shell 1@Shell 2

The previously prepared Ce-RENPs core@shell 1 cyclohexane solution was added into the Ce-RENPs shell 2 precursor directly and the temperature was raised to 60 °C for 15 min to remove the cyclohexane from the mixture. The reaction solution was then cooled down to 45 °C. The reaction mixture was heated to 110 °C for 15 min and 300 °C for 45 min under a nitrogen atmosphere. After naturally cooling it down to room temperature, the as-synthesized Ce-RENPs-SA core@shell 1@shell 2 nanoparticles in the solution were precipitated by adding 20 mL of acetone, collected by centrifugation at 10,000 rpm for 5 min, subsequently washed twice with a cyclohexane/acetone mixture (*v*/*v* = 1/3), and finally dispersed in 10 mL of cyclohexane for further use.

### 2.5. Synthesis of Ce-RENPs@SiO_2_-NH_2_

After 60 mg of Ce-RENPs was dispersed in cyclohexane (40 mL) and mixed with Igepal CO-520 (2 mL) and 25% NH_3_·H_2_O (0.32 mL) under sonication for 30 min, TEOS (0.03 mL) was added under stirring for 24 h to obtain Ce-RENPs@SiO_2_, which was further mixed with APTES (0.02 mL) to react for 24 h under stirring. The resulting Ce-RENPs@SiO_2_-NH_2_ was precipitated with acetone (10 mL), washed three times sequentially in aqueous HEPES solution and ethanol, and redispersed in DMF for future use. 

### 2.6. Synthesis of Ce-RENPs@SiO_2_-RB/FA

RB (30 mg, 0.03 mmol) was reacted with EDC (3 mg, 0.015 mmol) and NHS (3 mg, 0.065 mmol) in DMF (20 mL) for 2 h to obtain NHS-RB.

A total of 5 mL of the Ce-RENPs@SiO_2_-NH_2_ DMF solution was added with 1% NHS-RB (50 μL) and 1% NHS-PEG-FA DMF solution (100 μL) and stirred at room temperature and protected from light for 12 h. After centrifugation, the sample was washed twice with DMF and dispersed in 1 mL of DMF. 

### 2.7. Cell Culture

HeLa cells were cultured in Dulbecco’s modified Eagle’s medium (DMEM) supplemented with 10% FBS, 100 μg/mL of streptomycin, and 100 U/mL of penicillin–streptomycin at 37 °C in a humidified incubator containing 5% CO_2_ and 20% or 1% O_2_. 

### 2.8. ROS Detection

For extracellular ROS detection, a 0.1 mg/mL pretreated Ce-RENP@SiO_2_-RB/FA nanoprobe was mixed with 2.5 mM SOSG (200 μL), irradiated with a 980 nm light-emitting diode (LED) light at 1.5 W/ cm^2^, and the fluorescence intensity of SOSG was monitored at 488 nm over the irradiation time.

For intracellular ROS detection, the Hela cells were cultured with the RENP nanoprobe for 6 h and 50 μM SOSG for another 30 min at 37 °C, which were then exposed under a 980 nm LED light at 1.5 W/cm^2^ for different times to record the confocal laser scanning microscope (CLSM) fluorescence images.

### 2.9. In Vivo Imaging

BALB/c Nude mice were purchased from GemPharmatech (Nanjing, China), and all the in vivo experiments were performed in accordance with the NIH guidelines for the care and use of laboratory animals (NIH Publication no. 85-23 Rev. 1985) by qualified operators (Certificate Number of 202405A059). Ethical approval for the related animal experiments was obtained from the Animal Care and Use Committee of the Nanjing University of Chinese Medicine. To assess the feasibility of the Ce-RENPs-LBL@SiO_2_-RB/FA and Ce-RENPs-SA@SiO_2_-RB/FA nanoprobes for the in vivo NIR-II imaging, each type of nanoprobe was injected subcutaneously into separate sites on the same nude mouse. The in vivo NIR-II fluorescence images were obtained through the NIR-II in vivo imaging system with an 808 nm laser (10 W/cm^2^). The mice were sacrificed after the in vivo imaging was completed and all experimental methods were in accordance with the Helsinki Declaration.

### 2.10. Characterization

The transmission electron microscopic (TEM) and high-angle annular dark-field scanning transmission electron microscopy (HAADF-STEM) images were captured on a JEM-2100 transmission electron microscope (JEOL Ltd., Tokyo, Japan). Dynamic light scattering (DLS) was conducted on the ZetaPlus 90 Plus/BI-MAS (Brookhaven, NY, USA). Zeta potential analysis was conducted on a Nano-Z Zetasizer (Malvern, UK). The crystal structures of the obtained materials were characterized by X-ray diffraction (XRD) patterns and were performed on an Ultima IV 285 X-ray powder diffractometer (Rigaku Co., Tokyo, Japan). The NIR-II images were captured by an IVIS Lumina XR III in vivo imaging system (PerkinElmer, Waltham, MA, USA). The upconversion luminescence (UCL) spectra and down-shifting luminescence (DSL) NIR-II fluorescence spectra were detected by a FL spectrometer F980 (Edinburgh Instruments, Edinburgh, UK) under external 808 nm and 980 nm laser excitations. The cell images were captured on the NCF950 CLSM (Novel, Ningbo, China). The in vitro NIR-II FL imaging was performed under a home-built microscope equipped with a 790 nm laser diode (Changchun New Industries Optoelectronics, Changchun, China) and a thermoelectric cooling two-dimensional InGaAs camera (NIRvana 640, 640 × 512 pixels; Princeton Instruments (Trenton, NJ, USA), detecting range 900–1700 nm).

## 3. Results and Discussion

### 3.1. Synthesis Strategies and the Interface Characteristics 

**Figure 2:**
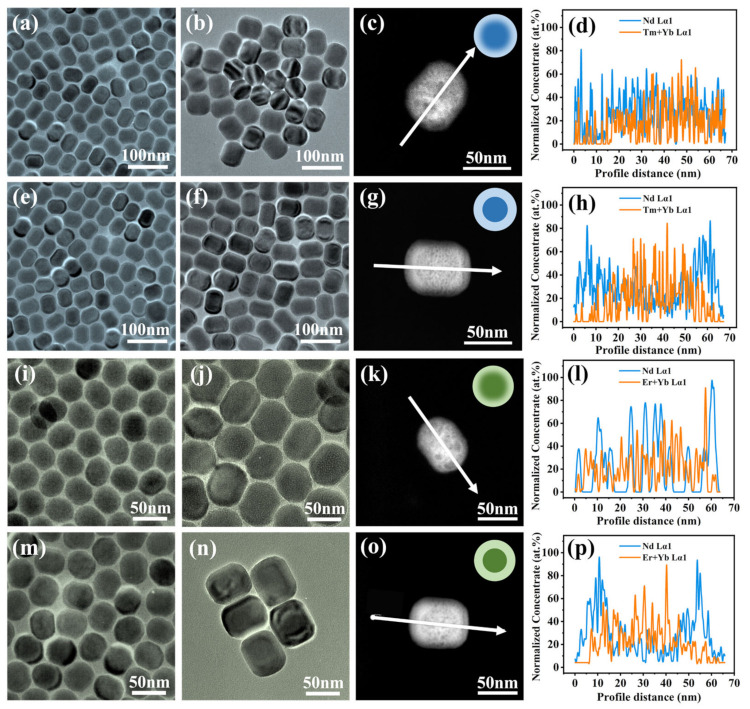
Characterization of the as-synthesized core–shell Tm-RENPs. TEM image of as-synthesized Tm-RENPs-LBL: (**a**) core, (**b**) core@shell; Tm-RENPs-SA: (**e**) core, (**f**) core@shell; Er-RENPs-LBL: (**i**) core, (**j**) core@shell; Er-RENPs-SA: (**m**) core, (**n**) core@shell. HAADF-STEM images of (**c**) Tm-RENPs-LBL, (**g**) Tm-RENPs-SA, (**k**) Er-RENPs-LBL, and (**o**) Er-RENPs-SA. Chemical concentration profiles of (**d**) Tm-RENPs-LBL, (**h**) Tm-RENPs-SA, (**l**) Er-RENPs-LBL, and (**p**) Er-RENPs-SA. The concentration profiles were obtained from the EDXS line; white arrows indicate the EDXS scan direction.

Due to the partial dissolution of the core during shell growth, which leads to interfacial blending at the core–shell boundary, we hypothesize that the shell injection method within the synthesis strategy results in varying elemental distributions at the interfaces of RENPs. To validate this hypothesis, we examined the interfacial distribution of RENPs synthesized through two different shell addition strategies using the conventional 808 nm excitation system (specifically Tm-RENPs and Er-RENPs, as shown in Figure S1). The RENPs were produced using the improved one-pot successive LBL protocol and the SA growth method. These RENPs exhibit nearly monodispersed characteristics with a uniform size distribution in each sample, as depicted in the transmission electron microscopy (TEM) image (Figure 2).

The initial investigation that focused on the Tm-RENPs revealed that the morphology and size of the Tm-RENPs synthesized using both methods were largely identical. The TEM images (Figure 2b,f) showed that the Tm-RENPs had a short rod-like shape with a size of 67.2 ± 1.2 nm (Figure S6), and the core measured 53.5 ± 1.3 nm (Figure 2a,e and Figure S6). Similarly, the Er-RENPs displayed a short rod-like morphology with a size of 65.3 ± 2.5 nm (Figure 2j,n) and a core size of 43.1 ± 1.1 nm (Figure 2i,m and Figure S6). From the TEM images, it was evident that the RENPs synthesized via the SA method had a clear core–shell interface (Figure 2f,n), whereas the RENPs synthesized using the LBL strategy had an almost invisible interface (Figure 2b,j). We hypothesize that the variation in the contrast of RENPs is due to the differing composition of rare earth elements in the core and each shell. The LBL strategy, which involves the co-growth of two precursors (with some core materials not fully reacting), tends to result in a contrast transition at the interface, making the interface less discernible.

We conducted further elemental analysis experiments to validate the aforementioned hypothesis. To achieve a spatial and chemical resolution of the interface quality of the RENPs, we employed the high-angle annular dark-field (HAADF)-STEM technique to characterize the RENP samples. The experimental results showed that the synthesis strategies using LBL or SA can successfully modulate the interface between the core and shell from being diffuse to sharp. Energy dispersive X-ray spectroscopy (EDXS) spectra were acquired successively along a line passing through the center of individual particles, as indicated by the arrow in the HAADF-STEM images (Figure 2, third column). The corresponding composition profiles (Figure 2, last column) were derived from the quantified EDXS spectra. It is important to emphasize that the chemical profiles of the shell (i.e., Nd, blue line) and core (i.e., Yb/Er or Yb/Tm, orange line) elements shown in Figure 2 (right column) were obtained after processing the compositions (extracted from the quantified EDXS spectra).

The concentration profiles (Figure 2 last column) and elemental mapping (Figure S6) similarly indicated that, in comparison to the LBL strategy, the core–shell interface of the RENPs synthesized via the SA strategy was sharper, resulting in a diminished diffusion of rare earth ions into the adjacent layer. The EDXS mapping and line scan analyses consistently revealed the distributions of lanthanide ions (especially the high amounts of Y, Yb, and Nd) in different regions of a single nanocrystal (Figure S6).

The LBL method might explain this phenomenon, as it requires repeated injections of shell precursors into a high-temperature core reaction environment. The extended exposure to 300 °C during shell growth likely causes parts of the core to disintegrate. As a result, rare earth ions were released from the dissolved core mix with the unripened shell precursors, which then re-deposited and grew on the original core surface. These findings provide evidence for severe nanoparticle dissolution under the tested conditions, aligning well with Hudry et al.’s speculation based on local chemical and structural analyses of core−shell RENPs [37]. In contrast, the improved SA strategy involves adding the entire preformed core to the shell precursor in a single step, followed by a rapid temperature increase until the reaction is complete, effectively preventing core disintegration. 

**Figure 3:**
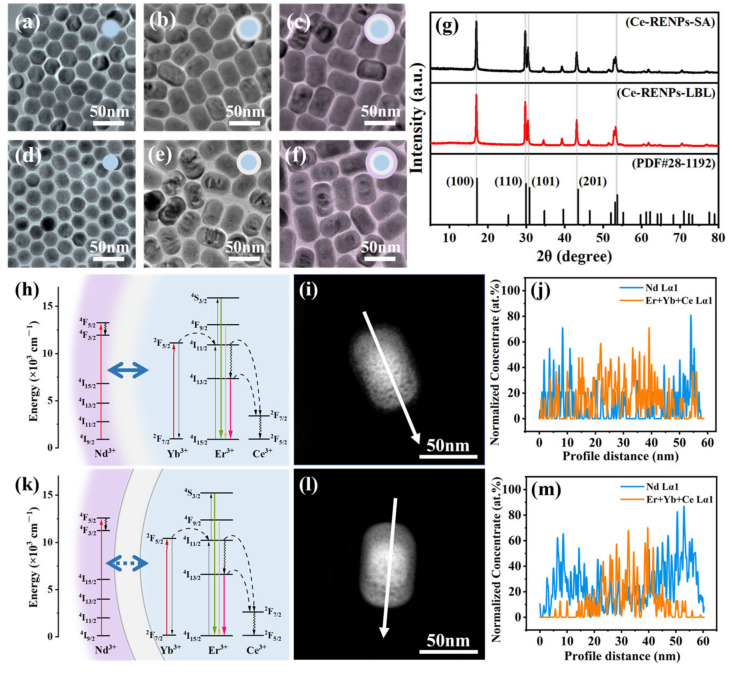
Characterization of the as-synthesized core–shell–shell Ce-RENPs. TEM image of as-synthesized Ce-RENPs-LBL (**a**) core; (**b**) core@shell 1; (**c**) core@shell 1@shell 2. TEM image of Ce-RENPs-SA: (**d**) core; (**e**) core@shell 1; (**f**) core@shell 1@shell 2. (**g**) XRD patterns of Ce-RENPs-LBL and Ce-RENPs-SA. Energy transfer diagram of (**h**) Ce-RENPs-LBL and (**k**) Ce-RENPs-SA. HAADF-STEM images of (**i**) Ce-RENPs-LBL and (**l**) Ce-RENPs-SA. Chemical concentration profiles of (**j**) Ce-RENPs-LBL and (**m**) Ce-RENPs-SA. The concentration profiles were obtained from the EDXS line; white arrows indicate the EDXS scan direction.

We then synthesized core–multi-shell structured Ce-RENPs (Figure 3) to extend the comparison of these two strategies to RENPs with multilayered shell configurations. The Ce-RENPs core–shell–shell samples exhibited a short rod morphology (Figure 3c,f) with average diameters of 59 nm and 62 nm (Figure S6), respectively. The X-ray diffraction (XRD) characterization of these nanoparticles shows a typical hexagonal phase (Figure 3g). A comparison of the measured XRD data with the standard hexagonal phase confirms identical peaks. All the diffraction peaks matched well with the standard peak positions of the hexagonal phase of Na(Y_0.57_Yb_0.39_Er_0.04_)F_4_ materials (JCPDS No. 28-1192), indicating that the as-prepared Ce-RENPs are highly crystallized.

The HAADF-STEM images and concentration profiles of the core–multi-shell structured Ce-RENPs revealed consistent trends. These data provide compelling evidence that the synthesis strategy has a pronounced effect on the quality of the core–shell interfaces. Furthermore, it underscores that the SA strategy can also partially mitigate interfacial blending between successive shells.

### 3.2. Interface Clarity and Energy Transfer 

**Figure 4:**
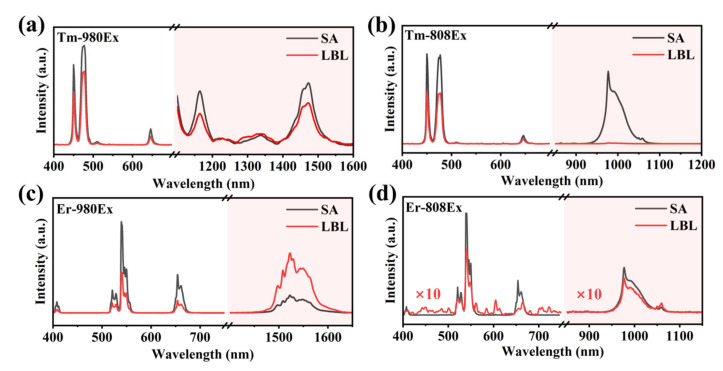
Fluorescence spectra of Tm-RENPs synthesized by two strategies under (**a**) 980 nm laser excitation and (**b**) 808 nm laser excitation. Fluorescence spectra of Er-RENPs synthesized by two strategies under (**c**) 980 nm laser excitation and (**d**) 808 nm laser excitation. The red shaded part is the DSL spectrum detected by the NIR detector.

We subsequently examined the effect of interface quality on the fluorescence properties and energy transfer in the RENPs synthesized via two different methods by directly measuring their fluorescence spectra. The UCL and DSL spectra of Tm-RENPs and Er-RENPs were then analyzed under 808 nm and 980 nm laser excitation at a power density of 10 W cm^−2^, respectively (Figure 4).

Due to the low absorption cross-section of Nd^3+^ ions in the shell at 980 nm, the shell’s contribution to energy transfer into the core is minimal, leading to negligible spectral differences in the overall UCL and DSL of the RENPs. However, the diffuse interface between the Tm-RENPs-LBL core and shell allows Nd^3+^ ions to diffuse into the core, facilitating direct interaction between Nd^3+^ and Tm^3+^. This interaction complicates the 4f energy levels between Tm^3+^ and Yb^3+^, causing energy loss. Consequently, the overall fluorescence emission intensity of the Tm-RENPs-LBL with a diffuse interface is reduced by approximately 23% compared to the Tm-RENPs-SA with a clear interface (Figure 4a).

Under 808 nm excitation, Nd^3+^ acts as a sensitizer, benefiting from the high absorption coefficient of Nd^3+^ ions at this wavelength and the efficient Nd→Yb non-radiative resonant energy transfer [38]. This transfer involves the migration of excitation energy from Nd^3+^ to Yb^3+^. At the characteristic emission peak of Yb^3+^ at 980 nm, the emission peak intensity of the Tm-RENPs-SA is significantly higher than that of LBL, indicating that the absorbed energy by Nd^3+^ is fully transferred to Yb^3^ and subsequently to Tm^3+^, enhancing the UCL at 450 nm and 488 nm (Figure 4b). In contrast, the Tm-RENPs-LBL undergoes relaxation (there exists a significant amount of non-beneficial energy exchange between Tm and Nd for UCL [39]), resulting in a substantial energy loss during the transfer from Nd^3+^ to Yb^3+^, thereby reducing the UCL emission intensity at the Tm excitation level.

Although the size and morphology of Er-RENPs are maintained (Figure 3c,f), upon 980 nm excitation, the UCL of the Er-RENPs-SA is stronger than that of the Er-RENPs-LBL, while the DSL at 1530 nm is weaker in the Er-RENPs-SA compared to the Er-RENPs-LBL. The DSL emission at 1523 nm, dominated by the ^4^*I*_13/2_→^4^*I*_15/2_ transition of Er^3+^, is significantly reduced by about 43% [40]. In contrast, the UCL at 650 nm and 540 nm decreased by 57% (Figure 4c). These observations can be attributed to the interface diffusion in the Er-RENPs-LBL, leading to partial contact between the Er in the core and the Nd in the shell. This contact increases the probability of multiphoton relaxation processes, promoting DSL over UCL. In the Er-RENPs core–shell nanoparticles, the migration of Er^3+^ from the core to the shell can induce emission quenching due to the energy transfer from the activators in the inner core to surface defects [24,38].

Similar to the Tm-RENPs, at 808 nm excitation, both the UCL and DSL of the Er-RENPs-SA are stronger than those of the Er-RENPs-LBL. At 808 nm excitation, The Yb^3+^ ion is used to extract the excitation energy from the sensitizer Nd^3+^ through interionic cross-relaxation [(^4^*F*_3/2_)_Nd_, (^2^*F*_7/2_)_Yb_]→[(^4^*I*_9/2_)_Nd_, (^2^*F*_5/2_)_Yb_], and the good overlaps of the Er^3+^: ^4^*I*_11/2_→^4^*I*_13/2_ emission with the Nd^3+^: ^4^*I*_9/2_→^4^*I*_13/2_ absorption and the Er^3+^: ^4^*I*_13/2_→^4^*I*_15/2_ emission with the Nd^3+^: ^4^*I*_9/2_→^4^*I*_15/2_ absorption grant an efficient energy back-transfer from the metastable Er^3+^ excited levels to the ^4^*I*_J_ levels of Nd^3+^ [41], followed by excitation-energy migration over the Yb sublattice and finally entrapment by the activator ions (Er^3+^) that are embedded in the inner core. An enhanced energy transfer from Nd^3+^ to Yb^3+^ in the Er-RENPs-LBL results in a weaker UCL and DSL compared to the Er-RENPs-SA. The clearer interface in the Er-RENPs-SA reduces energy loss, leading to nearly a ten-fold enhancement in UCL and DSL emission yields (Figure 4d). 

In summary, the efficiency of energy transfer within RENPs, influenced by interface clarity, is critical for the differences in UCL and DSL. This clarity is pivotal in preventing energy from undergoing irregular relaxation that could lead to fluorescence quenching, especially given the complex and rich energy transfer processes among rare earth ions.

**Figure 5:**
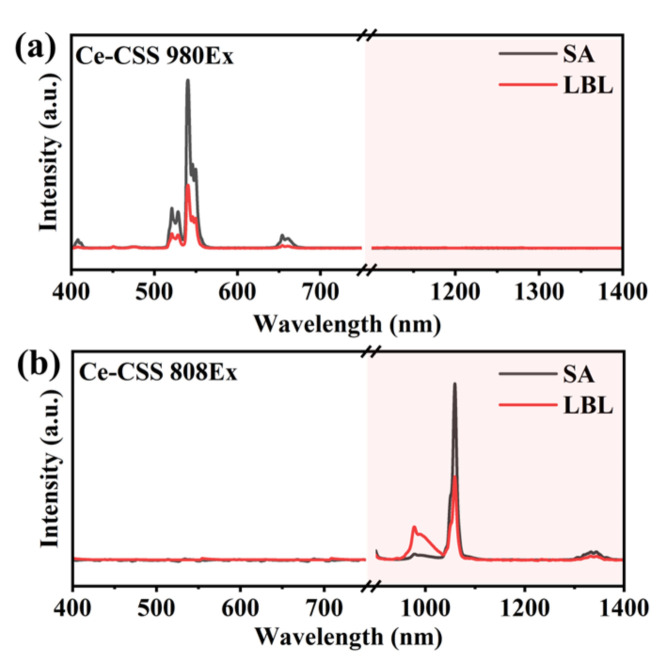
Fluorescence spectra of Ce-RENPs synthesized by two strategies under (**a**) 808 nm and (**b**) 980 nm excitation in cyclohexane at a power density of 10.0 W cm^−2^. The red shaded part is the DSL spectrum detected by the NIR detector.

Building on the impact of synthesis strategies on interface energy transfer, we utilized these insights to develop a multi-shell structure for orthogonal emission in RENP systems, resulting in the construction of Ce-RENPs (Figure S1). In the case of Ce-RENPs, doping with Ce^3+^ modulated the intermediate 4f–4f transitions of Er^3+^, thereby enhancing luminescence at DSL [5,42]. The first shell of NaYF_4_ is optically inert to the excited state ions and can prevent energy transfer from Nd^3+^ to interior lattice defects. The second shell, containing 5% Nd^3+^ ions, harvested 808 nm light and emitted at 1060 nm (Figure 5b), achieving orthogonal emission. This orthogonal emission structure in Ce-RENPs allows UCL and DSL to be independently generated by 808 nm and 980 nm excitation, respectively, without mutual interference.

Upon 980 nm laser excitation, the UCL intensity at 540 nm for the Ce-RENPs-SA is markedly enhanced compared to the Ce-RENPs-LBL (Figure 5a). This enhancement is likely due to the intact interface of the Ce-RENPs-SA, which enables an optically inert shell 1 of a specific thickness to effectively isolate the active core containing Yb from the outermost layer with Nd, thus reducing the probability of inward energy transfer.

Additionally, under 808 nm excitation, some electrons in the ^4^*F*_7/2_ excited state relax non-radiatively to the ^4^*F*_3/2_ excited state and then undergo radiative transition back to the ground state ^4^*I*_11/2_, emitting 1060 nm NIR-II fluorescence, which can be used for NIR imaging [43]. The emission spectrum from the DSL indicates that the Ce-RENPs-LBL has a lower emission at 1060 nm compared to the Ce-RENPs-SA, while the characteristic emission of Yb^3+^ at 980 nm is higher in the Ce-RENPs-LBL than in the Ce-RENPs-SA. This further corroborates the notion that the diffuse interface in the Ce-RENPs-LBL facilitates energy transfer from Nd to inner Yb, whereas a clear interface effectively prevents this transfer in the Ce-RENPs-SA. 

### 3.3. The Orthogonality of Luminescence and the Thickness of the Isolation Layer 

We observed that the inert isolation layer, shell 1, might hinder the energy transfer between Nd and Yb in Ce-RENPs. To address this, we optimized the thickness of shell 1 (d: from 1.0 to 8.1 nm) for both strategies and then characterized the orthogonal emission spectra of these particles. 

**Figure 6:**
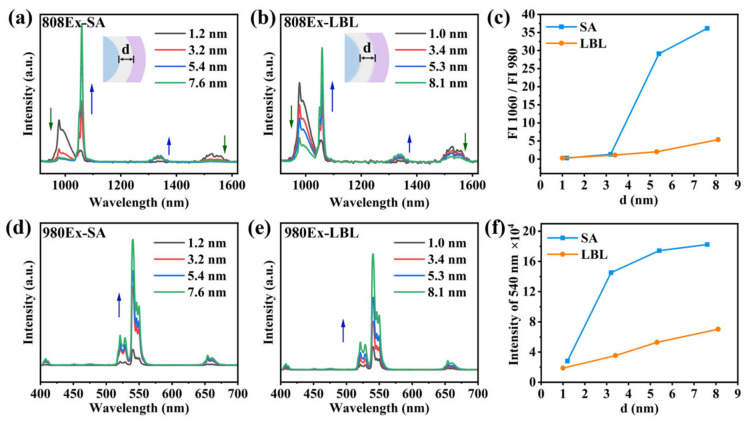
DSL spectra of (**a**) Ce-RENPs-SA and (**b**) Ce-RENPs-LBL at 808 nm excitation. (**c**) The intensity ratio of 1060 nm emission to 980 nm emission of Ce-RENPs-SA and Ce-RENPs-LBL under 808 nm laser excitation varies with the thickness of shell 1. UCL spectra of (**d**) Ce-RENPs-SA and (**e**) Ce-RENPs-LBL at 980 nm excitation. (**f**) The intensity of 540 nm emission of Ce-RENPs-SA and Ce-RENPs-LBL under 980 nm laser excitation varies with the thickness of shell 1. The blue arrow shows that the fluorescence intensity increases with the increase of shell 1 thickness, and the green arrow vice versa.

Under circumstances where the size and thickness of shell 1 are comparable, we measured the energy transfer efficiency by the ratio of fluorescence intensities at 1060 nm and 980 nm under 808 nm excitation, providing further insights into orthogonal emission performance. For Ce-RENPs synthesized using the SA strategy, the orthogonal emission was significantly enhanced when the thickness of shell 1 exceeded 5 nm. At a shell 1 thickness of around 8 nm, the energy transfer between Er^3+^ and Yb^3+^ within the core was almost entirely shielded, resulting in the orthogonal performance being 7 times higher than that of the Ce-RENPs-LBL (Figure 6c). When excited at 980 nm, the UCL of the nanoparticles synthesized using both strategies increased with the thickness of shell 1. At an equal shell 1 thickness, the Ce-RENPs-SA exhibited a superior UCL (Figure 6f). The minimized ion mixing due to a sharper interface in the SA strategy’s synthesized particles allows for achieving the desired orthogonal effect with a thinner interface thickness. This leads to smaller particles with brighter luminescence—advantageous for biological applications of multifunctional core–multi-shell structures.

### 3.4. ROS Generation and Detection of Ce-RENPs@SiO_2_-RB In Vitro and In Vivo

**Figure 7:**
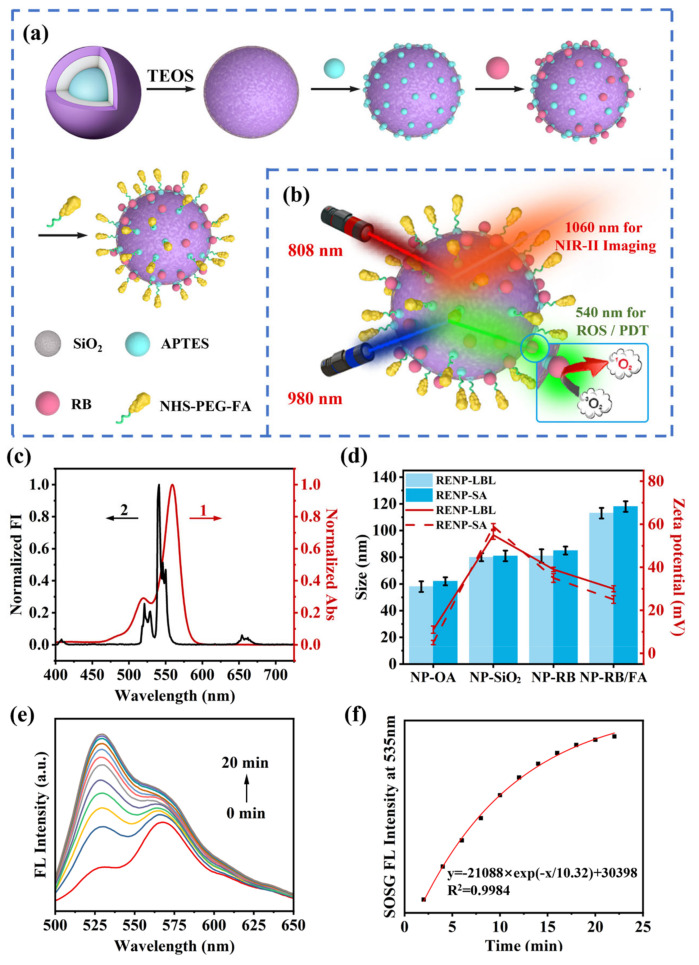
(**a**) Schematic diagram illustrating the modification process of Ce-RENPs@SiO_2_-RB/FA; (**b**) Ce-RENPs-RB/FA probes with dual-functional NIR-II in vivo imaging and ROS response and their corresponding UCL/DSL emissions; (**c**) UCL of the Ce-RENPs and the absorption of the RB; (**d**) hydrodynamic diameters and zeta potentials of Ce-RENPs-OA, Ce-RENPs@SiO_2_-NH_2_, Ce-RENPs@SiO_2_-RB, and Ce-RENPs@SiO_2_-RB/FA. Fluorogenic interactions between SOSG and ^1^O_2_, generated by photoirradiation of Ce-RENPs@SiO_2_-RB; (**e**) fluorescence emission spectra of RB and SOSG before irradiation and a mixture of RB and SOSG before and at different time points after 980 nm irradiation; (**f**) dependence of the fluorescence intensity on 980 nm irradiation time.

To enhance the biocompatibility and target specificity of Ce-RENPs, which have oleic acid ligands on their surface, a SiO_2_ layer was coated onto the Ce-RENPs and subsequently modified with amino groups to produce Ce-RENPs@SiO_2_-NH_2_. This modification enabled the efficient conjugation of Rose Bengal (RB), which emits in the 500−600 nm range and overlaps with the Ce-RENPs’ emission peak at 540 nm (Figure 7c), facilitating ROS generation for PDT. Furthermore, to further enhance cell targeting, folic acid (FA) was conjugated to the nanoprobe via amide bonds (Figure 7a). The resulting Ce-RENPs@SiO_2_-RB/FA exhibited a continuous size increase to 118.2 ± 2.4 nm (nanoparticles with diameters exceeding 150 nm are not conducive to in vivo therapy) with a zeta potential decrease to 31.7 ± 1.3 mV (Figure 7d).

Upon 980 nm excitation, Ce-RENPs emit at 540 nm, which activates RB and generates ROS. The produced ROS reacts with SOSG, serving as an indicator, resulting in an intense green fluorescence at 535 nm under 488 nm excitation [44]. The fluorescence intensity at 535 nm increases with the concentration of ROS. By mixing Ce-RENPs-RB with SOSG and subjecting the mixture to 808 nm LED illumination for 0 to 20 min, the fluorescence spectra under 488 nm excitation revealed a continuous increase in ROS production with increasing illumination time, indicating full activation of RB (Figure 7e).

**Figure 8:**
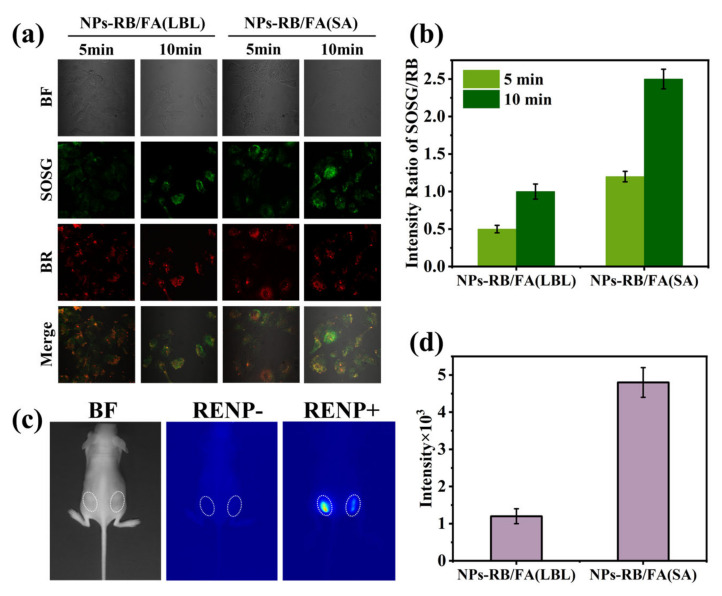
(**a**) Confocal fluorescence imaging of co-cultured HeLa cells with 50 μg/mL Ce-RENPs-RB/FA nanoprobes synthesized by the LBL/SA strategy and 50 μM SOSG under 980 nm excitation for 5 min and 10 min; (**b**) intensity ratio of confocal fluorescence between SOSG and RB; (**c**) NIR-II fluorescence imaging of subcutaneously injected mice treated with Ce-RENPs-SA (left circle) and Ce-RENPs-LBL (right circle) under 980 nm laser irradiation; (**d**) the NIR-II fluorescence imaging intensity values of Ce-RENPs-LBL/SA in mice.

The confocal fluorescence imaging results of co-cultured HeLa cells with 50 μg/mL Ce-RENPs-RB/FA nanoprobes, synthesized via the LBL/SA strategy, and 50 μM SOSG under 980 nm excitation for 5 min and 10 min (Figure 8a), respectively, indicate that the Ce-RENPs-SA-RB achieves a superior ROS generation effect (Figure 8b). After 10 min of 980 nm irradiation, the amount of ROS produced is 2.1 times higher than that of the Ce-RENPs-LBL-RB.

### 3.5. Enhanced NIR-II Imaging for SA Strategy

Another orthogonal channel has also reaped the benefits provided by the SA strategy. The Ce-RENPs-RB/FA was injected subcutaneously into mice, followed by NIR-II fluorescence imaging to capture the fluorescent images (Figure 8c). The findings indicated that the NIR-II fluorescence emission intensity of the Ce-RENPs-SA nanoprobes for the in vivo NIR-II imaging was 4.3 times higher than that of LBL. These observations collectively validate the immense potential of RENPs with a defined interface in biomedical applications (Figure 8d).

An increased ROS concentration signifies enhanced PDT treatment efficacy. A stronger NIR-II emission improves the diagnostic efficiency for deep-tissue pathologies. Consequently, the SA strategy aids in developing superior probe systems while keeping the nanoparticle size suitable for in vivo experiments. 

## 4. Conclusions

This study investigates the impact of synthesis strategies on the interface quality of core–shell structured RENPs by comparing the diffusion degree of the interface between the core and shell using the LBL and SA methods. Overall, although the widely employed LBL synthesis strategy can rapidly produce RENPs through a single, continuous operation, it tends to result in fusion at the core–shell interface, increasing the likelihood of cation mixing. This may lead to potential cross-relaxation and surface quenching effects, which, in turn, reduce the efficiency of energy transfer and further diminish the fluorescence emission intensity of RENPs. In contrast, our improved SA strategy can significantly form core–shell structured RENPs with a clear interface—a characteristic that is also applicable to core–multi-shell structures—and enhances the orthogonal emission of multifunctional RENPs with multiple shell layers. Moreover, as this strategy can markedly reduce interfacial diffusion, a thinner shell layer can achieve the desired luminescent effect, which is beneficial for the synthesis of smaller and brighter RENPs.

## Data Availability

Data are contained within the article.

## Supplementary Materials

The following supporting information can be downloaded at: https://www.mdpi.com/article/10.3390/nano14161326/s1. Figure S1. Schematic diagram of the structures of Tm-RENPs, Er-RENPs, and Ce-RENPs. Figure S2. Schematic diagram of Ce-RENPs-LBL synthesis strategies with different shell 1 thicknesses. Figure S3. Schematic diagram of Ce-RENPs-SA synthesis strategies with different shell 1 thicknesses. Figure S4. High-resolution TEM image and elemental mapping of the (a) Er-RENPs-LBL and (c) Er-RENPs-SA nanocrystals. The EDXS spectra of (b) Er-RENPs-LBL and (d) Er-RENPs-SA. Figure S5. High-resolution TEM image and elemental mapping of the (a) Ce-RENPs-LBL and (c) Ce-RENPs-SA nanocrystals. The EDXS spectra of (b) Ce-RENPs-LBL and (d) Ce-RENPs-SA. Figure S6. The size distributions of (a) Tm-RENPs-LBL, (b) Tm-RENPs-SA, (c) Er-RENPs-LBL, (d) Er-RENPs-SA, (e) Ce-RENPs-LBL, and (f) Ce-RENPs-SA.

## Abbreviations

RENPs	Rare Earth Nanoparticles
LBL	Layer-by-Layer
SA	Seed-Assisted
NIR	Near Infrared
PDT	Photodynamic Therapy
ROS	Reactive Oxygen Species
TEM	Transmission Electron Microscopy
HAADF-STEM	High-angle annular dark-field scanning transmission electron microscopy
EDXS	Energy dispersive X-ray spectroscopy
XRD	X-ray diffraction
UCL	Upconversion luminescence
DSL	Down-shifting luminescence
Tm-RENPs	Tm^3+^-doped RENPs
Tm-RENPs-LBL	Tm-RENPs synthesized by LBL strategy
Tm-RENPs-SA	Tm-RENPs synthesized by SA strategy
Er-RENPs	Er^3+^-doped RENPs
Er-RENPs-LBL	Er-RENPs synthesized by LBL strategy
Er-RENPs-SA	Er-RENPs synthesized by SA strategy
Ce-RENPs	Ce^3+^-doped RENPs
Ce-RENPs-LBL	Ce-RENPs synthesized by LBL strategy
Ce-RENPs-SA	Ce-RENPs synthesized by SA strategy
NHS	N-hydroxy succinimide
EDC	1-ethyl-(3-dimethyllaminopropyl) carbodiimide hydrochloride
HEPES	2-[4-(2-hydroxyethyl)-1-piperazinyl] ethanesulfonic acid
DMF	N,N-dimethylformamide
APTES	3-aminopropyl triethoxysilane
RB	Rose Bengal
FA	Folic acid
SOSG	Singlet oxygen sensor green reagent
CLSM	Confocal laser scanning microscope
LED	Light-emitting diode

## Appendix A

**Table A1:** The types of RENPs and the composition of the core and each shell.

Type of RENPs	Core Composition	Shell 1 Composition	Shell 2 Composition
Tm-RENPs	NaYF_4_: 0.5% Tm, 30% Yb	NaYF_4_: 10% Yb, 30% Nd	-
Er-RENPs	NaYF_4_: 2% Er, 20% Yb	NaYF_4_: 5% Nd	-
Ce-RENPs	NaYF_4_: 20% Ce, 2% Er, 20% Yb	NaYF_4_	NaYF_4_: 5% Nd
