# Supplementary material for: Core–Shell Interface Engineering Strategies for Modulating Energy Transfer in Rare Earth-Doped Nanoparticles
Source: Nanomaterials (Basel). 2024 Aug 7;14(16):1326. doi: 10.3390/nano14161326 (PMC11357452; doi:10.3390/nano14161326)
Supplement: Supplementary file 1 [file nanomaterials-14-01326-s001.zip › nanomaterials-3138754-supplementary.pdf]

Supporting Information

Core–shell Interface Engineering Strategies for Modulating Energy Transfer in Rare Earth Nanoparticles

Zhaoxi Zhou [1], Yuan Liu [1], Lichao Guo [2], Tian Wang [1], Xinrong Yan [1], Shijiong Wei [1], Dehui Qiu [1], Desheng Chen [1], Xiaobo Zhang [1,*] and Huangxian Ju [1,*]

[1] State Key Laboratory of Analytical Chemistry for Life Science, School of Chemistry and Chemical Engineering, Nanjing University, Nanjing, 210023, China
[2] School of Pharmacy, Nanjing University of Chinese Medicine, Nanjing 210023, China
* Correspondence: xhzxb@nju.edu.cn; hxju@nju.edu.cn

SUPPLEMENTARY FIGURES

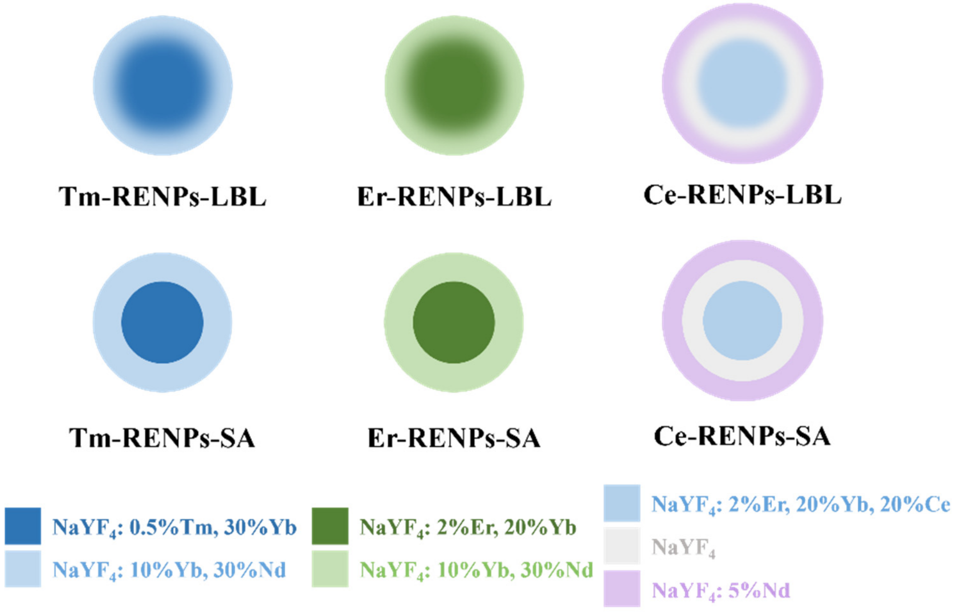

Figure S1. Schematic diagram of the Structures of Tm-RENPs, Er-RENPs, and Ce-RENPs.

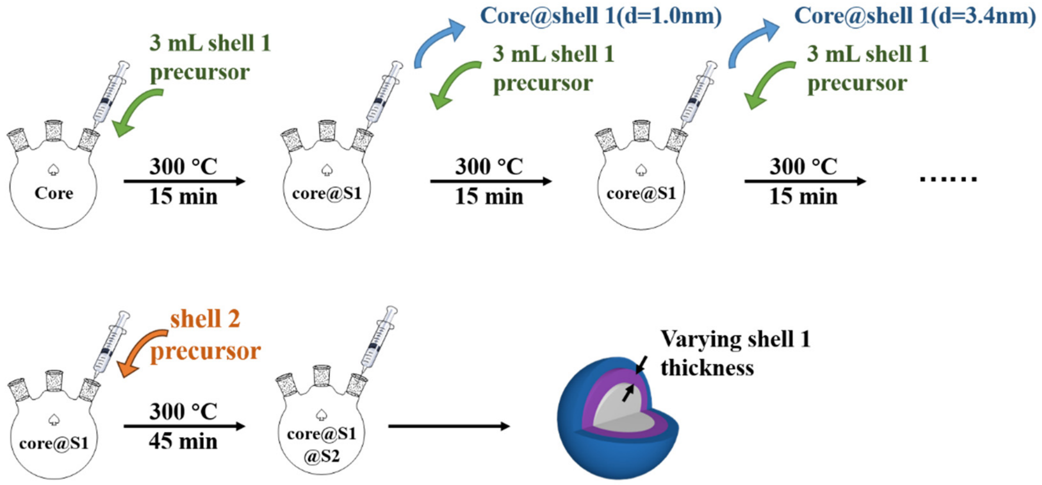

Figure S2. Schematic diagram of Ce-RENPs-LBL synthesis strategies with different shell 1 thicknesses.

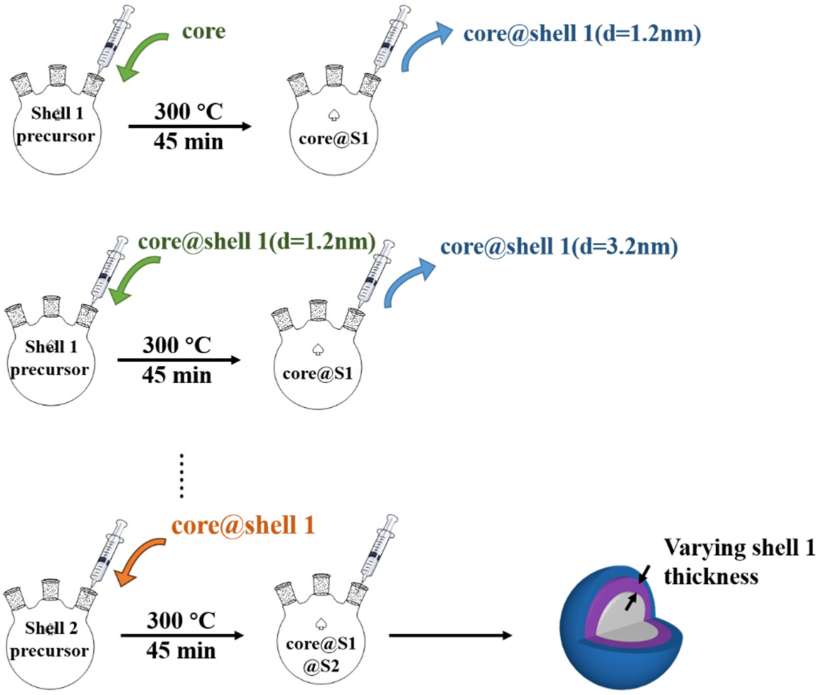

Figure S3. Schematic diagram of Ce-RENPs-SA synthesis strategies with different shell 1 thicknesses.

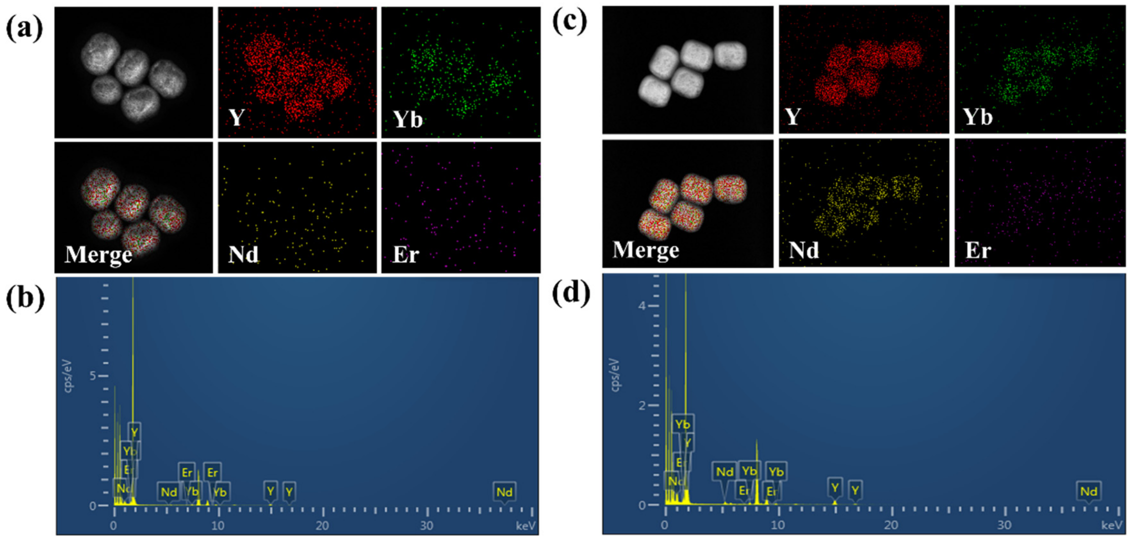

Figure S4. High-resolution TEM image and elemental mapping of the (a) Er-RENPs-LBL; and (c) Er-RENPs-SA nanocrystal. The EDXS spectrum of (b) Er-RENPs-LBL and (d) Er-RENPs-SA.

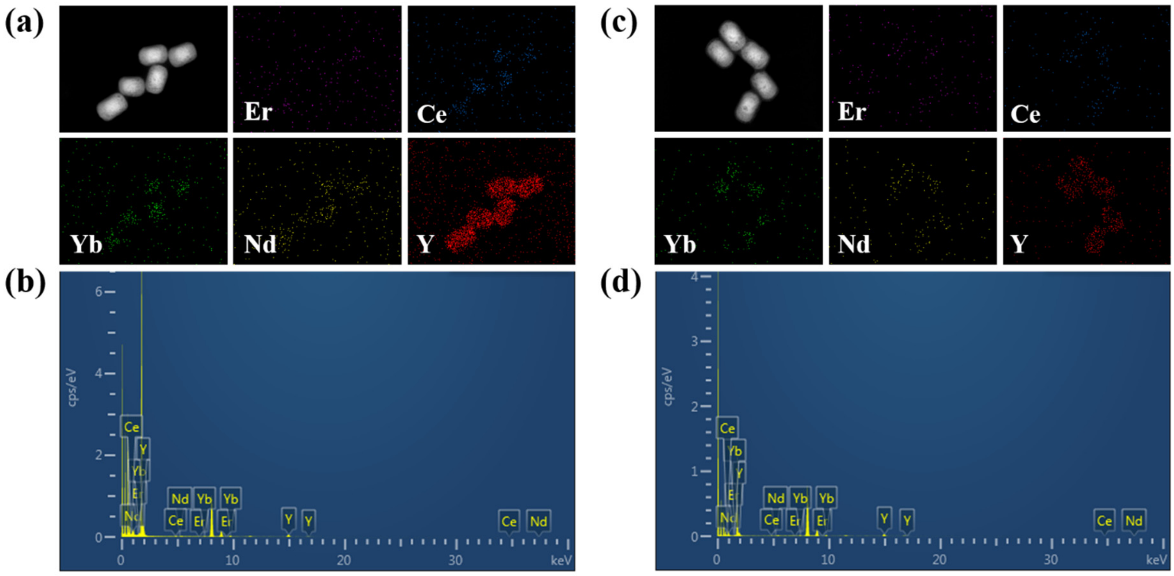

Figure S5 High-resolution TEM image and elemental mapping of the (a) Ce-RENPs-LBL and (c) Ce-RENPs-SA nanocrystal. The EDXS spectrum of (b) Ce-RENPs-LBL and (d) Ce-RENPs-SA.

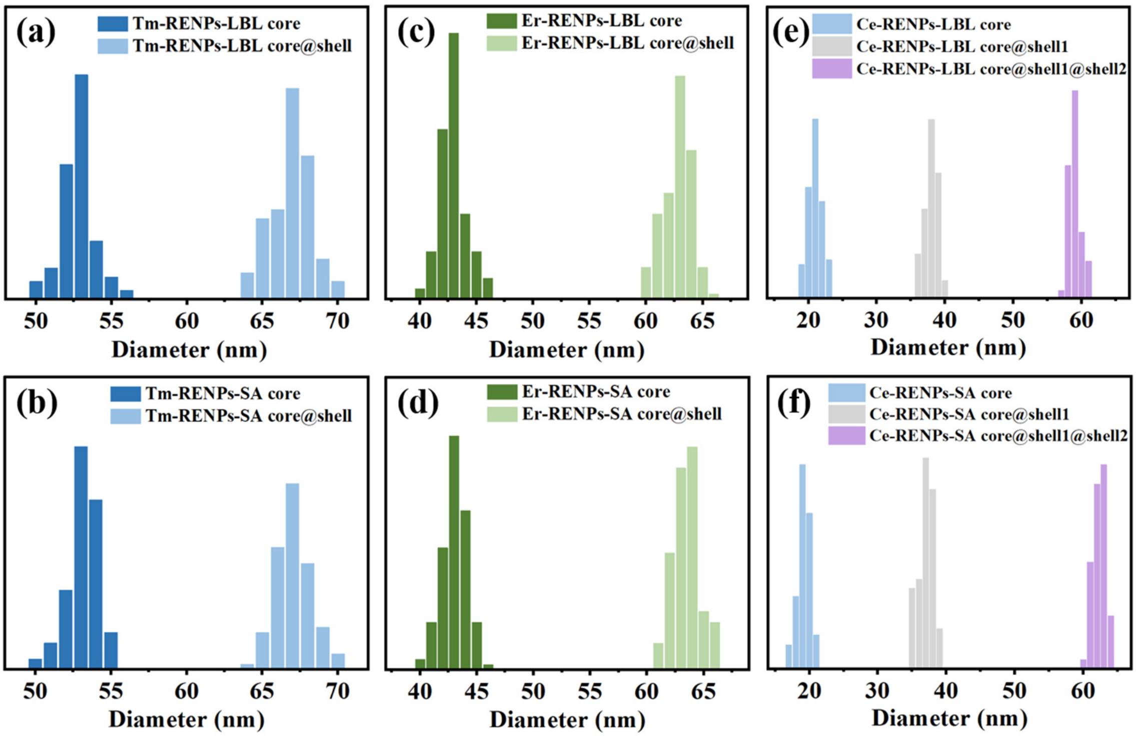

Figure S6. The size distributions of (a) Tm-RENPs-LBL; (b) Tm-RENPs-SA; (c) Er-RENPs-LBL; (d) Er-RENPs-SA;. (e) Ce-RENPs-LBL and (f) Ce-RENPs-SA.
